# Provision of Reproductive Healthcare to Women with Disabilities: A Survey of Obstetrician–Gynecologists' Training, Practices, and Perceived Barriers

**DOI:** 10.1089/heq.2018.0014

**Published:** 2018-08-01

**Authors:** Laura H. Taouk, Michael F. Fialkow, Jay A. Schulkin

**Affiliations:** ^1^ Research Department, The American College of Obstetricians and Gynecologists (ACOG), Washington, District of Columbia.; ^2^ Department of Psychology, American University, Washington, District of Columbia.; ^3^ Department of Obstetrics and Gynecology, University of Washington School of Medicine, Seattle, Washington.

**Keywords:** obstetrician–gynecologists, survey, health disparity

## Abstract

**Purpose:** The purpose of this study was to document current awareness, attitudes, and training regarding the care of women with disabilities by obstetrician–gynecologists (ob-gyns) and explore barriers that may explain observed discrepancies in care.

**Methods:** One thousand ob-gyns, including 500 members of the Collaborative Ambulatory Research Network (CARN), were surveyed on practice accessibility, training, awareness, barriers, beliefs, comfort, challenges, practices, contraceptive counseling, and preconception/pregnancy counseling.

**Results:** CARN, 49.0%, and non-CARN, 19.4%, members completed the survey for an overall response rate of 33.9%. Most respondents indicated feeling “somewhat” (57.5%) or “very” (21.9%) aware of the special healthcare needs of women with disabilities. Only 17.2%, however, received any information or training on the provision of healthcare to women with disabilities. Eighty-one percent agreed somewhat or strongly that women with disabilities are less likely to receive comprehensive reproductive healthcare. Respondents who provided contraceptive counseling (94.3%) initiated it with women of reproductive age who did not have a disability more frequently than those who had a disability. Finally, only 19.3% felt “definitely” adequately equipped to manage the pregnancies of women with disabilities.

**Conclusion:** Women with disabilities require reproductive healthcare no less than women without disabilities; however, the evidence consistently identifies disparities. This study suggests that while ob-gyn providers are aware of these issues, they lack adequate training and resources to provide equal care.

## Introduction

A disability can be defined as “a physical or mental impairment that substantially limits one or more major life activities.”^1^ More than 53 million adults in the United States reported a disability in 2013; mobility (13.0%) and cognitive (10.6%) disabilities were most common.^2^ Enacted in 1990 to promote equal access to a range of services, the Americans with Disabilities Act (ADA) prohibits discrimination against persons with disabilities in healthcare settings, such that accessible facilities, aids for effective communication, and modified procedures to address special needs are mandated.^1^

Evidence is accumulating that women with disabilities receive healthcare screening less often than is recommended. Women with physical and cognitive disabilities are screened for breast cancer and cervical cancer less frequently than women without disabilities.^3–5^ Even after controlling for demographic and health-related characteristics, women with disabilities are found to be less likely to be up-to-date with mammograms, Pap tests, pelvic examinations, and clinical breast examinations.^6–11^ Data further suggest the more severe the disability, the less likely women are to be screened for breast and cervical cancer.^3,9,12,13^ In addition to lower screening rates, women with disabilities also report lower receipt of family planning services, including contraceptive counseling, and concern regarding physician preparedness to handle their pregnancies.^14–16^

Healthcare providers likely face structural, informational, and attitudinal challenges to caring for women with disabilities.^17,18^ Differences in health service utilization and insurance coverage do not explain apparent discrepancies in screening between women with and without disabilities.^4,10,19^ Furthermore, women with disabilities are less likely to receive a doctor's recommendation for screening, suggesting that structural and/or clinical factors underlie observed differences.^4,10^ Barriers to the provision of care may include physically inaccessible facilities, lack of communication aids, inadequate appointment lengths, and lack of provider knowledge regarding disabilities and modified screening practices.^3,20,21^ In addition, women with disabilities could be perceived as less likely to be sexually active and, consequently, healthcare providers may make inaccurate assumptions about their reproductive healthcare needs.^20,21^

Obstetrician–gynecologists (ob-gyns) are critical to the provision of effective healthcare for women. While discrepancies in care are well documented, reports on ob-gyns' perspectives regarding the provision of care to women with disabilities are relatively limited (e.g., small sample size, narrow scope).^22^ To address this, we surveyed a nationally representative sample of ob-gyns, belonging to the American College of Obstetricians and Gynecologists (ACOG), regarding the care of women with physical disabilities and intellectual or developmental disabilities (hereafter abbreviated as I/DD). The aims of this study were to (1) document current awareness, attitudes, and training regarding the care of women with disabilities (2) as well as the perceived educational and clinical barriers that may explain observed discrepancies in care.

## Methods

### Measures

A six-page questionnaire was developed and revised based on pretesting with five clinically active ob-gyns. The first section of the survey assessed practice accessibility, training, awareness, barriers, and beliefs about the provision of care to women with disabilities. Sections two and three focused on comfort, challenges, and practices associated with the care of women with physical disabilities (defined as “conditions that limit physical functioning, mobility, dexterity, and/or stamina; e.g., muscular dystrophy, amputation, or sensory impairments”) and I/DD (defined as “conditions that can affect language, learning, reasoning, problem solving, adaptive behaviors, and/or independent living; e.g., Down syndrome, fragile X syndrome, and cerebral palsy”). Subsequent sections assessed issues related to contraceptive counseling and preconception/pregnancy counseling. Demographic questions were included.

### Procedures

The University of Washington Institutional Review Board determined the study to be of exempt status, due to the use of minimal risk survey procedures. Data collection began in November 2016. A random sample of 1,000 ACOG Fellows, 500 of whom belonged to the Collaborative Ambulatory Research Network (CARN), were invited to participate. CARN comprised Fellows who volunteer to participate in survey studies, and samples are found to be demographically representative of the greater ACOG membership.^23^ Ob-gyns were e-mailed links to the online questionnaire, along with information for informed participation, through the survey platform Qualtrics. Reminders were sent to nonresponders who had not opted out. In February, paper copies of the survey and prepaid return envelopes were mailed to nonresponders. Finally, an abbreviated version of the survey, which retained demographics and most items from the first three sections, was mailed to nonresponders. Data collection ended in May 2017.

### Participants

Of those invited to participate, 49.0% of CARN and 19.4% of non-CARN members completed the survey for an overall response rate of 33.9% (*n*=322). Responses were excluded if the physician was no longer in practice (*n*=1) or left at least 50% of the survey blank (*n*=13) for a final sample of 308 ob-gyns. Half completed the survey online (53.9%; 46.1% on paper), and most completed the full-length version (87.0%; 13.0% abbreviated version). Sample demographics are described in Table 1. Males had been in practice longer (*m*=28.6±8.4) than females (*m*=19.0±9.3; *t*=9.03, *p*<0.001). Few ob-gyns (3.6%) had a disability, while 27.9% had a close friend or family member with a disability. CARN members were slightly more likely to practice in urban areas and midsized towns than non-CARN participants (χ^2^=10.00, *p*=0.040), however, other demographic differences were not found. Data were analyzed in aggregated form.

**Table 1.** **Sample Demographics (*N*=308)**

Years in practice postresidency	22.8±10.1	Age	54.3±10.2
Gender (%)		Practice setting (%)	
Female	59.7	Ob-gyn partnership/group	28.2
Male	38.6	University faculty and practice	18.5
Ethnicity/race (%)		Hospital or clinic	16.6
White	78.2	Multispecialty group	14.6
Black or African American	6.5	Solo private practice	13.6
Asian	6.5	HMO/staff model	3.2
Multiracial	2.9	Military/government	2.3
Hispanic or Latino	2.3	Practice location (%)	
Primary medical practice (%)		Suburban	36.0
General ob-gyn	64.9	Urban, noninner city	23.4
Gynecology only	24.7	Urban, inner city	19.2
Obstetrics only	8.4	Midsized town (10,000–50,000)	12.3
		Rural	6.8

HMO, health maintenance organization; ob-gyn, obstetrician–gynecologist.

### Statistical analyses

Data analysis was conducted using a personal computer-based software package (IBM SPSS Statistics^®^ 23.0; IBM Corp©, Armonk, NY). Categorical response options endorsed by <10% of the sample were collapsed; incremental, unidirectional response options were evaluated as continuous variables. Relationships between categorical variables were evaluated using chi-square tests. Relationships between continuous variables were evaluated using Pearson correlations, while group differences in continuous variables were evaluated using independent-samples and paired *t*-tests and ANOVAs. Linear regression models were also used to explore continuous and dichotomous predictors of continuous outcomes. All tests were considered significant at *p*<0.05, and valid percentages are reported.

## Results

### Awareness and training

Most ob-gyns indicated feeling “somewhat” (57.5%) or “very” (21.9%) aware of the special healthcare needs of women with disabilities. Greater awareness was related to more years in practice (*F*=6.15, *p*=0.002); those who felt “very aware” were also more likely to have a close friend or relative with a disability (χ^2^=13.82, *p*=0.001) or received information or training (χ^2^=19.73, *p*<0.001). Only 17.2% of ob-gyns had received *any* information or training on the provision of healthcare to women with disabilities. Information or training on facilitating pelvic examinations for patients with mobility-limited physical disabilities (88.9%) and modifying communication approach for those with visual, hearing, or cognitive disabilities (71.1%) were most common; preventing autonomic reactions to an examination was least common (28.9%). Receipt of training/information was less likely for ob-gyns in private practice or a partnership/group (χ^2^=13.60, *p*=0.018), but unrelated to years in practice. Only 4.8% reported additional training would be “not beneficial at all.”

### Provision of care and barriers

Most ob-gyns (81%) agreed somewhat or strongly that “women with disabilities are less likely to receive comprehensive reproductive healthcare than women without disabilities.” Only 2.3% rated the provision of comprehensive healthcare for women with disabilities as “not challenging at all.” Barriers are listed in Table 2. Average barrier scores (α=0.794; *m*=1.71±0.42) were lower for ob-gyns whose practice was handicap accessible (e.g., ramps, elevators, wide doors; 94.4%; *t*=−2.52, *p*=0.012), had at least one examination room with adapted medical equipment to accommodate persons with disabilities (e.g., adjustable-height examination tables; 86.2%; t=−2.25, *p*=0.025), and had resources to communicate with patients with vision or hearing impairments (e.g., paperwork in Braille, ASL speaker; 42.2%; *F*=6.10, *p*=0.003). Ob-gyns in solo private practice or a partnership/group were more likely to endorse the starred items as major barriers (Table 2: χ^2^=21.37, *p*=0.019; χ^2^=20.17, *p*=0.028; χ^2^=27.76, *p*=0.002) and less likely to have an accessible examination room (χ^2^=44.70, *p*<0.001) or communication resources (χ^2^=82.62, *p*<0.001).

**Table 2.** **Barriers to the Provision of Care**

	Not a barrier (%)	Minor barrier (%)	Major barrier (%)
Inaccessible office location and equipment	76.3	18.8	4.2
Limited insurance reimbursement for extra time and care provided^*^	39.9	32.5	27.3
*Difficulty with positioning during examinations*	27.6	54.5	17.5
*Fear of autonomic dysreflexia or other autonomic reactions to the examination*	51.1	41.4	6.3
Fear of causing patients discomfort, pain, or embarrassment	56.5	36.0	7.1
Inadequate knowledge about specific disabilities and special needs^*^	31.5	54.2	14.0
*Uncertainty regarding appropriate sexual and reproductive recommendations*	56.3	34.7	9.0
Difficulty communicating with patients who have visual, hearing, or cognitive disabilities^*^	28.2	48.1	23.7
Uncertainty about decision-making capacities or consent to medical procedures with patients who have intellectual or developmental disabilities	28.6	50.0	21.4

Responses to the question stem: “in your practice, what are the barriers to the provision of healthcare for women with disabilities?” (*N*=308). Each item was rated as “not a barrier,” a “minor barrier,” or a “major barrier”. Italicized items were not included on the abbreviated survey (*N*=268). Starred items were more likely to be endorsed as major barriers by ob-gyns in private practice or a partnership/group.

### Comfort and confidence

**FIG. 1.**
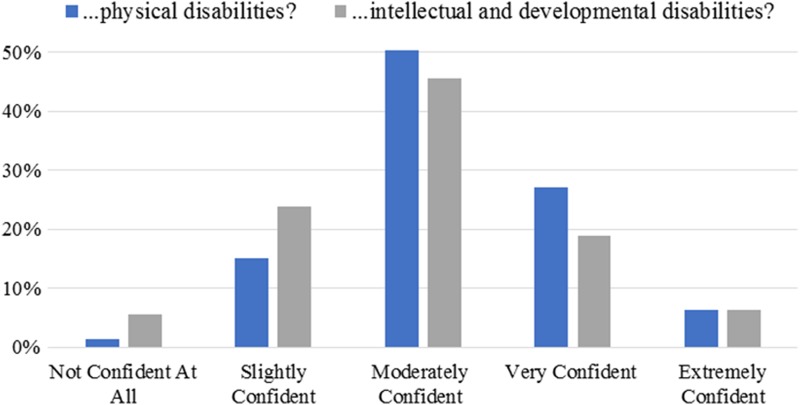
Confidence in ability to provide care to women with disabilities. Responses to, “how confident are you in your ability to provide appropriate healthcare for women with… [(1) physical disabilities; (2) intellectual and developmental disabilities],” were rated on a scale from “not confident at all” to “extremely confident” (*N*=304).

In a typical month, ob-gyns estimated they saw an average of 4.3 (±20.9) patients with a physical disability and 4.2 (±4.1) with an I/DD. For women with physical disabilities, most ob-gyns were “very comfortable” asking about gynecological, sexual, and reproductive history (68.5%); performing a pelvic examination (65.9%); performing a breast examination (80.0%); and managing sexual/reproductive care (58.0%). For women with an I/DD, fewer ob-gyns were “very comfortable” with these parts of well-women care (respectively: 50.0%, 46.7%, 58.4%, 44.7%). Greater average comfort ratings (α=0.913, *B*=0.26; α=0.936; *B*=0.41), receipt of information or training (*B*=0.23; *B*=0.29), and fewer barriers to the provision of care (*B*=−0.77; *B*=−0.67) predicted higher physician confidence in their ability to provide appropriate care for women with *physical* disabilities (*F*=29.88, *p*<0.001; *R*^2^=0.288) and I/DD (*F*=61.05, *p*<0.001; *R*^2^=0.380). Confidence ratings are shown in Figure 1. Years in practice and number of patients seen were not associated with comfort nor confidence providing care.

### Practices

For mobility-limited patients, most ob-gyns never (43.2%) or only sometimes (50.4%) performed examinations with women remaining in their mobility device. However, a portion of respondents indicated they “never” examined high-pressure areas of skin for decubitus ulcers or pressure sores (29.9%) nor recommend earlier screening for osteoporosis (27.9%) or cardiac health (41.2%). For patients with limited bodily sensation, 60.1% of ob-gyns reported they “never” provided information about alternative (nonsensation based) symptoms of breast and cervical cancer. If clinically indicated pelvic examinations could not be accommodated, most ob-gyns preferred to recommend an examination under sedation or anesthesia (34.7%) or an ultrasound (32.2%). For patients with an I/DD, some ob-gyns reported they “most of the time” or “always” primarily communicated with the patient's guardian (49.0%) and deferred to the guardian to make health decisions in situations of shared decision-making status (38.0%). Half (51.1%) reported mostly or always using supported decision-making strategies to assist patients in making decisions.

### Contraceptive counseling

**FIG. 2.**
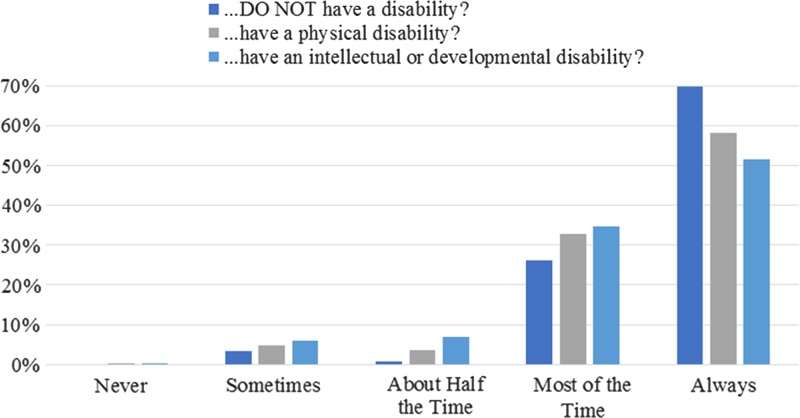
Frequency of initiating contraceptive counseling. Responses to, “in your current practice, how often do you initiate contraceptive counseling with women of reproductive age who…[(1) do not have a disability; (2) have a physical disability; (3) have an intellectual or developmental disability],” were rated on a scale from “never” to “always.” Items were answered by full-length survey respondents who provided contraceptive counseling (*N*=244).

**FIG. 3.**
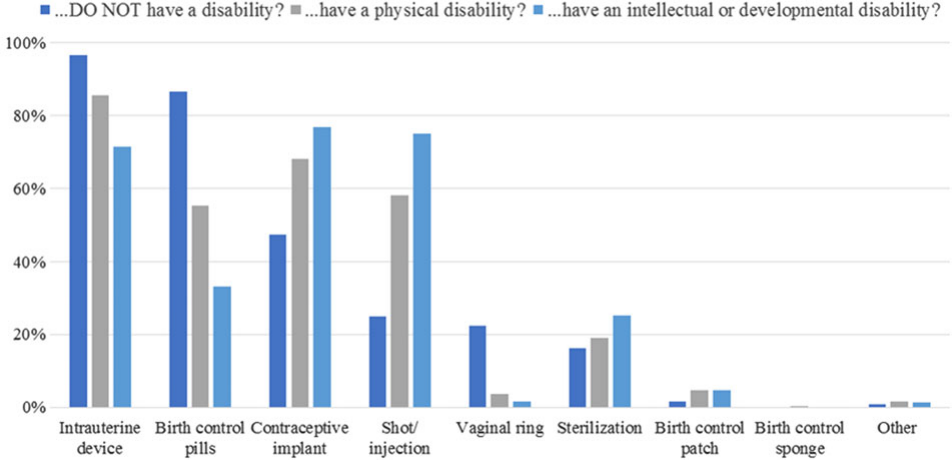
Top three contraception recommendations. Responses to, “please rank the top three types of contraception that you most often recommend for patients who…[(1) do not have a disability; (2) have a physical disability; (3) have an intellectual or developmental disability]. Items were answered by full-length survey respondents who provided contraceptive counseling (*N*=241). Since many respondents did not provide numbered rankings (e.g., writing #2 next to their second choice), responses were recoded to reflect options that were selected as top three recommendations.

Ob-gyns who provided contraceptive counseling (94.3%) initiated it with women of reproductive age who did not have a disability more frequently than those who had a physical disability (*t*=5.68, *p*<0.001) or an I/DD (*t*=7.03, *p*<0.001; Fig. 2). However, most “strongly” disagreed that women with a physical disability (59.8%) or an I/DD (64.3%) were less likely to require contraceptive counseling. Barriers are listed in Table 3. Lower barrier scores (*m*=2.0±0.5; α=0.878) related to more frequent initiation of contraceptive counseling for women with physical disabilities (*r*=−0.14, *p*=0.025) and I/DD (*r*=−0.17, *p*=0.009) and feeling more adequately equipped to manage contraceptive care of women with disabilities (30.2% “definitely yes,” 56.3% “probably yes,” 13.4% “probably or definitely not”; *F*=20.80, *p*<0.001). Ob-gyns who were aware of *any* guidelines on contraceptive counseling for women with disabilities (24.9%) were more likely to feel adequately equipped (χ^2^=10.53, *p*=0.005) and rank intrauterine devices as a top three contraception recommendation for women with physical disabilities (χ^2^=4.28, *p*=0.039) and I/DD (χ^2^=5.76, *p*=0.016; Fig. 3). Sterilization was more likely to be a top three recommendation for women with I/DD (*t*=−2.99, *p*=0.003), but not physical disabilities (*t*=−1.26, *p*=0.209), compared with women without disabilities.

**Table 3.** **Barriers to Contraceptive Counseling**

	Not a barrier (%)	Minor barrier (%)	Major barrier (%)
Determining whether patients require contraceptive counseling	56.3	39.2	4.1
Determining ability to independently and properly utilize contraception	24.1	49.0	26.9
Determining decision-making capacities for contraception and sex	20.8	49.0	29.0
Determining consent to irreversible means of contraception	17.1	46.5	36.3
Determining patient understanding of contraception risks and benefits	13.9	58.4	27.8
Determining patient understanding of STD or pregnancy prevention	13.5	58.8	27.8

Responses to the question stem: “in your practice, what are the barriers to providing contraceptive counseling for women with disabilities?” Each item was rated as “not a barrier,” a “minor barrier,” or a “major barrier.” Items were answered by full-length survey respondents who provided contraceptive counseling (*N*=245).

STD, sexually transmitted disease.

### Preconception and pregnancy counseling

Of the 90.0% of ob-gyns who counseled patients considering pregnancy, 29.5% “most of the time” or “always” referred patients with disabilities to a specialist. Those who provided preconception counseling most frequently emphasized potential difficulties with the pregnancy (93.5%), difficulties with labor and delivery (88.7%), and genetic effects, or consultation with a genetic counselor (84.8%). Potential resources to support the transition into pregnancy and parenthood were least frequently emphasized (54.5%). Only 19.3% of physicians felt “definitely” adequately equipped to manage the pregnancies of women with disabilities (52.4% “probably yes”; 20.2% “probably not”; 8.2% “definitely not”). Nearly all ob-gyns (92.2%) endorsed a need for more informational resources to help physicians guide women with disabilities through pregnancy, its management, and the transition into parenthood.

## Discussion

Women with disabilities require reproductive healthcare no less than women without disabilities; however, access to skilled and culturally competent care, where special needs are recognized and addressed, remains an obstacle. Although evidence points to persistent disparities in women's reproductive care, provider perspectives remain sparse in the literature. This study evaluated practicing ob-gyns' comfort, opinions, and practices regarding the provision of well-woman, gynecological, and obstetric healthcare to women with disabilities. Findings highlight barriers perceived by ob-gyns, as well as educational and structural needs, which may inform future efforts toward reproductive health equity.

Supporting evidence of disparities, 81% of ob-gyns in our sample believed women with disabilities are less likely to receive comprehensive reproductive healthcare.^3,4^ While most ob-gyns reportedly had handicap accessible practices, less than half had communication resources, and 71.8% endorsed “difficulty communicating with patients who have visual, hearing, or cognitive disabilities.” Although required by ADA, there is evidence to suggest many subspecialty practices cannot accommodate patients with mobility impairments and auxiliary aids/services are still lacking.^20,24,25^ Other common barriers (Table 2) implicate inadequate knowledge as a prominent challenge faced by physicians, consistent with prior findings.^26^

Nearly all ob-gyns indicated additional training would be at least slightly beneficial. Only 17.2% had received any information or training on the provision of healthcare to women with disabilities and far fewer on specific issues such as preventing autonomic dysreflexia, a potentially life-threatening condition that can occur during gynecological examinations of women with spinal cord injuries.^18^ Higher training rates have been found in a general physician sample (22.8%, 34.1%).^27^ The Accreditation Council for Graduate Medical Education (ACGME) indicates ob-gyn residents are “expected to demonstrate sensitivity and responsiveness to a diverse patient population, including … disabilities,” but didactic requirements are absent.^28^

Despite barriers and training deficits, most ob-gyns felt aware of the special healthcare needs of women with disabilities, and few felt uncomfortable with components of an examination for women with physical disabilities (≤6.5%) or I/DD (≤12.8%). Greater gynecologist discomfort was endorsed in a decade-old study (14–42%).^26^ While ob-gyns likely attempt to accommodate patients as best they can, some reported practices were concerning. For instance, when treating patients with mobility limitations, around one in three ob-gyns reportedly *never* examined high-pressure areas of skin for decubitus ulcers or pressure sores, which are common sources of increased pain and morbidity, nor recommended early bone density or cardiovascular disease screening, despite increased risk.^3,29,30^ In addition, only half of ob-gyns endorsed using supported decision-making strategies most or all the time for patients with I/DD, despite their utility in protecting patients' rights and assisting them in making health decisions.^31^

Regarding contraceptive counseling, it was reportedly initiated more frequently with nondisabled patients, even though few ob-gyns (<9%) indicated women with disabilities were less likely to require it. This discrepancy between reported practice and opinion could suggest ob-gyns harbor implicit stereotypes of women with disabilities as asexual, which leads to biased provision of care. Alternatively, or additionally, this discrepancy could be explained by nonattitudinal barriers. Except for “determining whether patients require contraceptive counseling,” each barrier was endorsed by more than 75% of the sample (Table 3). Counseling requires special considerations, such as ability to provide informed consent and independently utilize contraception, yet only one in four ob-gyns was aware of any guidelines on contraceptive counseling for women with disabilities.^18^ Although experts recommend against irreversible contraception, a substantial number of ob-gyns selected sterilization as one of the top three types of contraception they most often recommended for patients with physical disabilities (19.1%) or I/DD (25.3%; Fig. 3).^18,32^ Encouragingly, rank-ordered responses indicated sterilization was rarely ranked as the first-choice recommendation (physical disabilities: 0%, I/DD: 2.7%).

During preconception counseling, ob-gyns emphasized many potential patient concerns, but only half emphasized resources to support the transition into pregnancy and parenthood. Nearly all ob-gyns acknowledged a need for more informational resources to help physicians guide women with disabilities through pregnancy, its management, and the transition into parenthood, as previously proposed.^14^ Only 19.3% of ob-gyns felt “definitely” adequately equipped to manage the pregnancies of women with disabilities. This is consistent with patient reports that clinicians are ill equipped to manage their pregnancies effectively.^14^ Such findings are alarming, given evidence that women with disabilities, particularly I/DD, are at a greater risk for pregnancy and birth complications, as well as adverse pregnancy outcomes.^33–35^

The present study examined the provision of reproductive healthcare to women with disabilities in a representative sample of practicing ob-gyns, contributing much needed data on provider needs and perspectives. However, this study had several limitations. To begin with, the sample size was lower than hoped for, and therefore, results may not ideally represent practicing ob-gyns. Second, the survey was designed to obtain a general understanding of the issues for ob-gyns, since little is currently known. Complementary questions regarding the care of nondisabled women were not always included, constricting exploration of whether responses reflect disability-specific opinions, attitudes, comfort, and practices. Some terms (e.g., “accessible”) were not defined, which could have affected responses. Furthermore, it is possible that social desirability bias influenced responses to questions regarding opinions and attitudes. Self-reported practices may also differ from actual care delivered, and interpretation of reported practices is limited, as individualized, case-by-case considerations cannot be known. Considering physical disabilities and I/DD encompass a wide range of presentations, physicians potentially responded to questions with varying levels of disability severity in mind. Finally, questionnaire-based data preclude the possibility of drawing causal conclusions.

## Conclusion

This study is hopefully a first step toward improving quality of care for women with disabilities. Findings indicate training deficits, inadequate knowledge, and unawareness of guidelines could be significant barriers preventing ob-gyns from providing comprehensive reproductive healthcare to women with disabilities. Results also suggest ob-gyns' confidence in their ability to provide appropriate care could be improved by targeting training/information, practice barriers, and comfort with components of the examination that may require modified approach. Training physicians to meet the special (e.g., cervical and breast cancer screening) needs of women with disabilities is a federally recognized issue.^21^ Education and resources are needed to support physicians and patients with disabilities and promote comfort, respect, and safety.^18,32^

Going forward, it is imperative that educational initiatives, as well as clinical guidelines, are developed, empirically evaluated, and disseminated to reproductive healthcare practitioners. Materials, such as “The Toolbox” and ACOG's recorded slide program, are available to facilitate the implementation of care; however, their utilization, reach, and relevance to practitioners are presently unknown.^36,37^ Data are critically needed to “inform policy and program development regarding critical issues of health disparities and health equity,” and systemic change in healthcare delivery may ultimately be necessary.^38,39^ Our hope is that the data presented here will contribute to greater awareness of the barriers encountered by ob-gyns in their practice, informing future efforts toward the goal of equity in reproductive healthcare.

## Abbreviations Used

ACOG: American College of Obstetricians and Gynecologists
ADA: Americans with Disabilities Act
CARN: Collaborative Ambulatory Research Network
I/DD: intellectual or developmental disabilities
ob-gyns: obstetrician–gynecologists
